# Curdlan-Induced Significant Enhancement of Lipid Oxidation Control and Gelling Properties of Low-Salt Marine Surimi Gel Containing Transglutaminase and Lysine

**DOI:** 10.3390/gels11070535

**Published:** 2025-07-10

**Authors:** Wenhui Ma, Guangcan Liang, Qiliang Huang, Feng Ling, Weilin Pan, Yungang Cao, Miao Chen

**Affiliations:** 1 School of Basic Medicine, Hubei University of Medicine, Shiyan 442000, China; mawenhui@hbmu.edu.cn (W.M.); pwl503710110@163.com (W.P.); 2 Natural Food Macromolecule Research Center, School of Food Science and Engineering, Shaanxi University of Science and Technology, Xi’an 710021, China; 15903053270@163.com; 3 Linyi Jinluo Win Ray Food Co., Ltd., Linyi 276036, China; hql120@hotmail.com (Q.H.); lfwsl71@163.com (F.L.)

**Keywords:** curdlan, novel marine surimi gel, lipid oxidation, gel properties

## Abstract

In this study, curdlan was investigated as a substitute for egg-white protein, and the effects of different concentrations (0.2%, 0.4%, 0.6%, 0.8%, and 1.0%) on lipid oxidation and the physicochemical properties of a novel low-salt surimi gel containing transglutaminase (TGase) and lysine were evaluated. The results indicated that adding appropriate curdlan concentrations (0.2%–0.4%, especially 0.4%) significantly inhibited lipid oxidation in the surimi gel, achieving the highest L* and whiteness values. The fracture strength, WHC, hardness, and chewiness of the gel increased by 23.87%, 6.70%, 32.80%, and 13.49%, respectively, compared to the control gel containing egg-white protein (*p* < 0.05). At 0.4% curdlan, the gel also enhanced the crosslinking within the surimi, improved its resistance to shear stress, significantly increased the G’ value, and shortened the T_21_, T_22_, and T_23_ relaxation times, inhibiting the conversion of immobilized to free water in the gel and promoting a denser three-dimensional network structure. However, excessive curdlan amounts (0.6%–1.0%) led to a notable deterioration in the gel performance, causing a more irregular microstructure, the formation of larger cluster-like aggregates, and a negative effect on color. In conclusion, the combination of 0.4% curdlan with TGase and Lys is effective for preparing low-salt surimi products.

## 1. Introduction

“Miscellaneous marine fish” refers to non-cultured marine species commonly harvested as bycatch or regarded as low-value fish. Despite their abundance and high nutritional value, particularly in high-quality animal proteins and phospholipids, their commercial utilization is limited due to their small size, numerous bones, and low flesh yield. Surimi processing offers an effective strategy to increase its added value and improve resource efficiency. Surimi is a refined myofibrillar protein (MP) extracted from fish muscle using mechanical chopping, washing, refinement, and dehydration processes, followed by the addition of specific materials for grinding, shaping, heating, and cooling [1]. MP, as a typical salt-soluble protein, tends to aggregate into filaments under low salt concentrations (<0.3 M), which weakens protein–protein interactions and deteriorates the quality of surimi products [1,2]. In contrast, at high salt concentrations (>0.3 M), the aggregation of myosin is suppressed, promoting the unfolding of protein structures and the exposure of internal hydrophobic groups and sulfhydryl groups, thereby facilitating the formation of a compact three-dimensional network [3,4,5]. However, excessive sodium intake is positively associated with an increased risk of stroke and various chronic diseases [6].

To reduce the sodium content in meat products, two innovative strategies have recently been introduced to improve or sustain the gelation performance of low-salt surimi products. One strategy incorporates exogenous additives or sodium salt alternatives [7,8,9], while the other focuses on developing advanced processing techniques [10,11]. Transglutaminase (TGase) can catalyze the covalent crosslinking between glutamine and lysine (Lys) residues in proteins, thereby enhancing their gelation, emulsification, and other functional properties [12]. Notably, the effectiveness of TGase depends on the availability of lysine residues, and recent studies have shown that exogenous lysine can enhance TGase-induced crosslinking and promote gel formation in myofibrillar systems [13,14]. Egg-white protein is another widely used additive in surimi processing, contributing to improved gel strength and textural properties [15]. However, its high cost and adverse impact on product color limit its application in commercial production. Therefore, identifying cost-effective and functional alternatives is of both technological and economic importance.

Curdlan, a neutral microbial polysaccharide, has gained increasing attention due to its unique thermal gelling behavior and synergistic interaction with proteins. When heated to 55–65 °C, curdlan forms a reversible gel via hydrogen bonding between single-helix chains; at temperatures above 80 °C, it transitions to a stable triple-helix structure that forms strong, irreversible gels through hydrophobic interactions [16,17]. Xu et al. [18] found that the addition of thermoreversible curdlan or thermo-irreversible curdlan promoted the transition from α-helix to β-sheet structures, as well as enhanced hydrophobic interactions and disulfide bond formation, which contributed to the formation of a compact and uniform protein network structure. Zhu et al. [19] demonstrated that the integration of TGase and curdlan in a two-stage thermal process could form a double-network surimi gel with enhanced mechanical integrity and elasticity. However, these studies have predominantly focused on the effects of curdlan on gelation properties, particularly its interactions with proteins in conventional high-salt systems. In contrast, research on the role of curdlan in controlling lipid oxidation—especially under low-salt conditions—is extremely limited.

Therefore, we selected curdlan as a replacement for egg-white protein and studied its impact on the lipid oxidation and gel properties of a novel low-salt surimi gel containing TGase and lysine. The findings of this study contribute to the advancement of technologies to improve the gel properties of low-salt surimi while maintaining its safety, quality, and consumer acceptability, promoting the sustainable development of the surimi processing industry.

## 2. Results and Discussion

### 2.1. Lipid Oxidation

**Figure 1:**
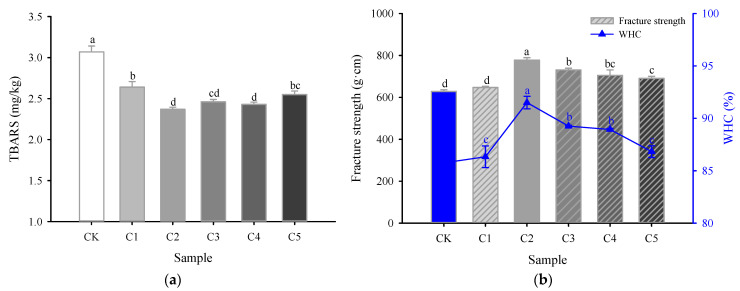
Effects of curdlan concentration on lipid oxidation (**a**), fracture strength (**b**), and WHC (**b**) in novel marine surimi gel samples. CK represents the novel marine surimi gel containing egg-white protein; C1, C2, C3, C4, and C5 represent the novel marine surimi gels in which egg-white protein is replaced by 0.2%, 0.4%, 0.6%, 0.8%, and 1.0% curdlan, respectively. Different letters (a–d) marking the same parameter group indicate significant differences (*p* < 0.05).

Surimi contains various oxygenases, leading to the inevitable oxidation and decomposition of certain substances, particularly polyunsaturated fatty acids, which result in the production of significant amounts of MDA. The latter is a secondary lipid oxidation product that interacts with thiobarbituric acid (TBA), generating reddish-brown compounds referred to as TBARS [20]. Typically, a higher TBARS content indicates a greater degree of fat oxidation [21]. The TBARS content in surimi gel was influenced by the incorporation of curdlan, as shown in Figure 1a, where the corresponding content in the CK group was approximately 3.07 mg/kg. Following curdlan was added, the TBARS content in the surimi gels initially declined and then rose; however, these values remained significantly lower than those observed in the CK group. Notably, when the added amount of curdlan was 0.4% (C2), the TBARS content of the surimi gel reached its minimum at 2.37 mg/kg, indicating that curdlan effectively inhibited the fat oxidation in surimi.

This inhibitory effect may be partly attributed to the interaction between curdlan and the oxidizable lipid fraction in surimi. According to previous studies, marine fish commonly used in surimi production contain a substantial proportion of polyunsaturated fatty acids, particularly eicosapentaenoic acid (EPA) and docosahexaenoic acid (DHA), which are highly prone to oxidative degradation [22,23]. These unsaturated fatty acids serve as the primary substrates for oxidation, leading to elevated MDA formation under pro-oxidant conditions. The incorporation of curdlan may reduce oxidative susceptibility by enhancing water-binding capacity, limiting oxygen diffusion, and promoting the formation of a denser gel matrix that restricts lipid mobility and enzymatic access.

### 2.2. Fracture Strength and Water-Holding Capacity

The fracture strength and WHC values are closely related to the quality characteristics of meat-based food products. As illustrated in Figure 1b, the addition of curdlan had a dose-dependent effect on the fracture strength of surimi compared to that of the CK group. In particular, the fracture strength of the composite surimi increased significantly as the curdlan concentration rose from 0.2% to 0.4% (*p* < 0.05). At a 0.4% curdlan concentration, the maximum fracture strength reached 788.02 g·cm, representing a 23.87% increase over the CK group (*p* < 0.05). This improvement is mainly due to the ability of curdlan to promote interactions between MP and absorb free water in the system during heating, thereby increasing the crosslinking density and improving gel performance [10]. It is also worth noting that curdlan is a high-molecular-weight polysaccharide, and its molecular weight may vary depending on the manufacturer and production batch. Such variability can influence its water-binding capacity and interaction with proteins, potentially leading to differences in optimal dosage across studies. For instance, Lu et al. [10] reported that incorporating 0.6% curdlan with magnetic field-assisted freezing significantly enhanced the fracture strength of Penaeus vannamei surimi. Additionally, combining curdlan with TGase in the composite surimi system promoted the formation of more crosslinked proteins, resulting in a denser gel matrix, similar to the findings of Hu et al. [24]. The higher effective concentration observed in their study may be partly attributed to differences in curdlan molecular characteristics or interaction dynamics under different processing conditions.

In contrast, when the curdlan concentration rose from 0.6% to 1.0%, the fracture strength of surimi exhibited a significant decline (*p* < 0.05). The negative effects at higher concentrations may arise from two factors [17]: (1) the strong water absorption and swelling capacity of curdlan can lead it to compete with protein for water molecules, disrupting the protein gelation; (2) an excess of curdlan during the protein self-assembly process can result in the growth of larger aggregates, which hinder the formation of the protein gel network.

As illustrated in Figure 1b, the WHC of the CK group was 85.76%. The addition of curdlan led to an initial increase followed by a decrease in the WHC of surimi. At a curdlan concentration of 0.4%, the WHC peaked at 91.50%, which was 6.70% higher than that of the CK group (*p* < 0.05). Hu et al. [17] observed similar results in their study on the effects of curdlan on the gel properties and molecular interactions of whey protein isolate gels. The increase in WHC indicates that higher amounts of water are retained or bound within the gel network structure. When heated to 90 °C, curdlan can absorb a substantial amount of water to form highly gelled, thermo-irreversible gels, thereby enhancing the interactions with myofibrillar proteins. This process facilitates the development of a dense, uniform network during thermal induction, effectively trapping a greater amount of water [17,24]. However, as the amount of curdlan continues to increase, the WHC of the surimi gel decreases significantly (*p* < 0.05). This suggests that an excess of curdlan may reduce the protein–water-binding sites, limiting the binding and interactions between surimi proteins and water molecules, and ultimately results in a lower WHC.

### 2.3. TPA

The sensory acceptability of meat products can be evaluated through texture analysis, making it a crucial parameter for assessing product quality. Five key indicators are used to monitor textural changes in samples: hardness (g), springiness, cohesiveness, chewiness (g), and resilience [25]. As shown in Table 1, the hardness of the surimi gel samples first increased and then decreased with the addition of curdlan, similar to the trend observed for the fracture strength. When curdlan was added at 0.4% concentration, the hardness reached its peak, exceeding that of the CK group by 32.80%. The springiness, cohesiveness, and resilience remained largely unchanged with increasing curdlan concentration (*p* > 0.05), while the chewiness followed a trend similar to that of the hardness. According to Petcharat and Benjakul [26], hydrocolloids (gellan) in surimi proteins likely function as binders or fillers, enhancing the texture properties of surimi gels by facilitating electrostatic interactions. Similarly, Zhao, Zhou, and Zhang [27] found that regenerated cellulose fibers integrate into the protein network, disrupting hydrogen bonds in α-helix conformations and causing a transition to β-sheet structures, significantly improving the hardness and viscoelasticity of MP gels. Additionally, the thermal gelation properties of curdlan, the catalytic activity of TGase, and the Lys-induced protein conformational changes all positively affect the textural properties of surimi gels. However, negative effects were observed when the added curdlan concentration exceeded 0.4%. This might be due to the excess curdlan absorbing higher water amounts for gelation, which prevents the complete formation of the surimi MP gel network [28]. Consequently, the catalytic activity of TGase is reduced, resulting in a deterioration of the textural properties of surimi gels.

### 2.4. Color

**Figure 2:**
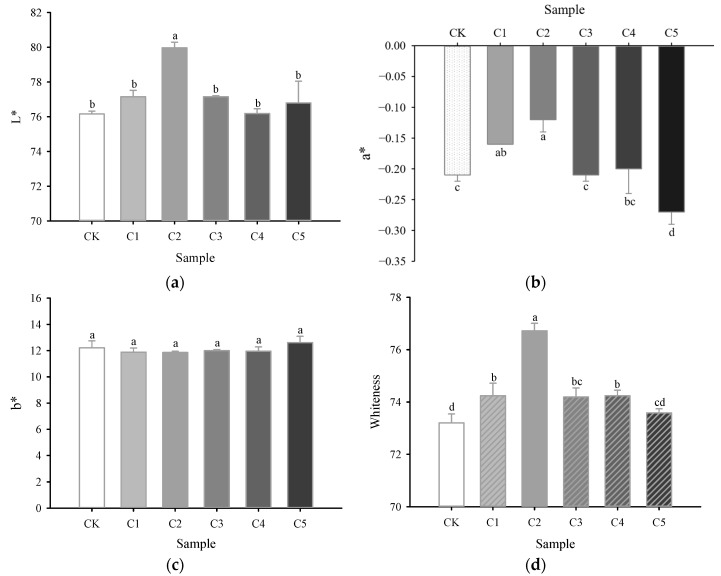
Effects of curdlan concentration on lightness (L*, (**a**)), redness/greenness (a*, (**b**)), yellowness/blueness (b*, (**c**)), and whiteness (**d**) of novel marine surimi gel. CK represents the novel marine surimi gel containing egg-white protein; C1, C2, C3, C4, and C5 represent the novel marine surimi gels in which egg-white protein is replaced by 0.2%, 0.4%, 0.6%, 0.8%, and 1.0% curdlan, respectively. Different letters (a–d) marking the same parameter group indicate significant differences (*p* < 0.05).

The color characteristics of surimi gel are primarily affected by the type and concentration of exogenous additives, with higher whiteness typically being more desirable to consumers [29]. Figure 2 illustrates the color changes in the novel marine surimi gel when varying amounts of curdlan replace egg-white protein. When the added curdlan concentration reached 0.4%, the L* (Figure 2a) and whiteness values (Figure 2d) of the surimi gel reached their peaks, which were 5.0% and 4.8% higher than those of the CK group, respectively, while the absolute value of a* (Figure 2b) significantly decreased (*p* < 0.05). This could be attributed to the gel-forming ability of curdlan, which enhances the WHC of the surimi gel (Figure 1b), thereby reducing the free water content at the surface, decreasing the dispersion of light, and increasing the reflectance [10]. Consistent with this observation, Mi et al. [29] reported that the inclusion of hydroxypropylated cassava starch, curdlan, and κ-carrageenan (HCK) could enhance the whiteness of silver carp surimi gel and improve product quality. However, as the added curdlan concentration increased from 0.4% to 1%, both the L* and whiteness values of the surimi gel significantly decreased, while the absolute value of a* significantly increased (*p* < 0.05). This suggests that excessive amounts of curdlan adversely affected the sensory properties of the novel low-salt marine surimi gel. No significant differences were found in the b* values across all sample groups (*p* > 0.05) (Figure 2c).

### 2.5. Rheological Properties

#### 2.5.1. Steady-State Shear Properties

**Figure 3:**
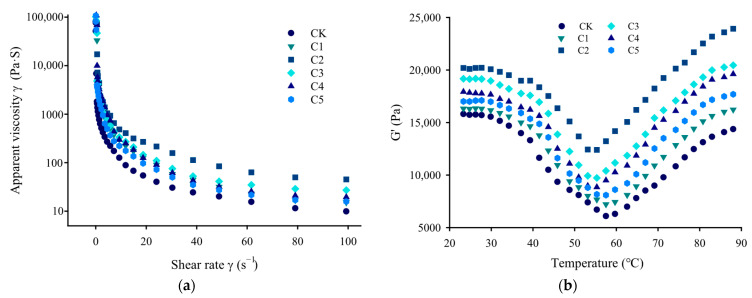
Apparent viscosity (η, (**a**)) and storage modulus (G’, (**b**)) during the heating of novel marine surimi samples treated with different amounts of curdlan. CK represents the novel marine surimi gel containing egg-white protein; C1, C2, C3, C4, and C5 represent the novel marine surimi gels in which egg-white protein is replaced by 0.2%, 0.4%, 0.6%, 0.8%, and 1.0% curdlan, respectively.

Regarding rheological properties, changes in the apparent viscosity of surimi reflect its internal structural crosslinking characteristics and resistance to deformation [30]. The steady shear curves in Figure 3a reveal that, with increasing shear rate, the apparent viscosity of all composite surimi samples decreased significantly, indicating shear-thinning behavior. In comparison to the control group (CK), the surimi samples in which curdlan replaced egg-white protein exhibited significantly higher apparent viscosity (*p* < 0.05), peaking at a 0.4% curdlan concentration. This suggests that curdlan enhanced the internal crosslinking, thereby improving the resistance to high shear stress [31]. Zhang, Chen, and Teng [32] also reported that curdlan significantly increased the apparent viscosity of MP across the whole variable-speed shear process. However, upon gradually increasing the curdlan concentration (0.6–1.0%), the apparent viscosity decreased, mirroring the trends observed for the textural properties of surimi gel (Table 1).

#### 2.5.2. Dynamic Elastic Properties During Gelation

Dynamic temperature sweep analysis effectively illustrated structural changes in the surimi samples at varying temperatures during the gelation transition process. The storage modulus (G’) is a key indicator of structural transitions in surimi gel, reflecting changes in solid properties, including elasticity and deformability [33]. As shown in Figure 3b, all samples exhibited similar dynamic temperature sweeping curves in the range of 20 to 90 °C. Specifically, as the temperature increased, the G’ curves of the novel marine surimi samples displayed two characteristic stages: an initial decrease followed by a gradual increase. This behavior is mainly due to the different denaturation temperatures of various surimi components [29]. For the CK group, the G’ value gradually decreased within the 20–57 °C temperature range. This decrease can be attributed to several factors [19]: (1) the degradation of surimi proteins by endogenous proteases, which disrupts the integrity of the gel network structure; (2) the breakage of hydrogen bonds between heat-induced protein molecules and the aggregation of MP; (3) the presence of Lys, facilitating the dissociation of actomyosin into myosin and actin. Subsequently, upon reaching the minimum temperature of ~57 °C, further heating (from 57 to 90 °C) resulted in a sharp rise in the G’ value. This increase is mainly attributed to the denaturation and crosslinking of the heavy chains of surimi myosin, which contribute to the development of a dense, uniform, and thermally irreversible gel network structure [4].

The rise in G’ reflects a significant improvement in gel fracture strength and elasticity [4]. Throughout the heating process, the G’ values of the curdlan-treated samples remained significantly higher than those of the CK group (*p* < 0.05), gradually increasing with the addition of curdlan, and reaching a maximum value at a 0.4% concentration, suggesting that curdlan enhances intermolecular interactions. However, as the curdlan concentration exceeded 0.4%, the G’ values decreased, possibly due to reaching a critical curdlan concentration in the surimi system, leading to a strong thermodynamic incompatibility that appeared as phase separation [25]. This trend aligns with variations in fracture strength, water-holding capacity, and texture characteristics presented in Figure 1a and Table 1.

Furthermore, incorporating curdlan reduced the critical temperature at the G’ minimum in the surimi sample. Specifically, the C2 group (0.4% curdlan) exhibited a critical temperature of 53 °C, which was 4 °C lower than that of the CK group. This decrease suggests that the curdlan treatment reduces the denaturation temperature of myosin light chains in surimi [2].

### 2.6. LF–NMR Relaxation

**Figure 4:**
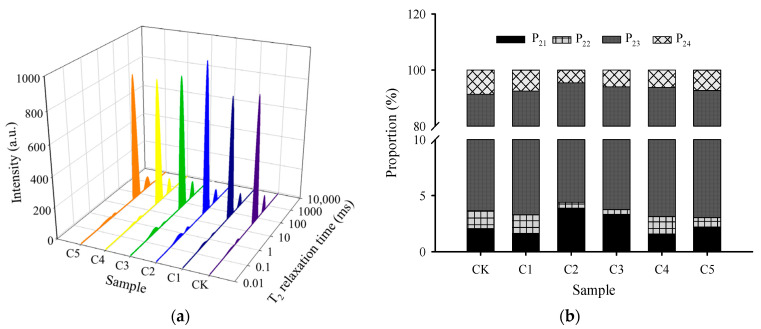
Effects of curdlan concentration on LF–NMR T_2_ relaxation curves (**a**) and relative content of different water states (**b**) in novel marine surimi gel samples. CK represents the novel marine surimi gel containing egg-white protein; C1, C2, C3, C4, and C5 represent the novel marine surimi gels in which egg-white protein is replaced by 0.2%, 0.4%, 0.6%, 0.8%, and 1.0% curdlan, respectively.

The water migration and distribution, as well as the interactions between water molecules and biopolymers in surimi gel systems, were assessed using LF–NMR [34]. By inverting the LF–NMR data, transverse relaxation time (T_2_) spectra were obtained, as shown in Figure 4a. These spectra revealed four distinct peaks, corresponding to different water states: The T_2_ spectra of all samples were fitted with four peaks, corresponding to strongly bound water (T_21_, 0.1–1 ms), weakly bound water (T_22_, 1–10 ms), immobilized water (T_23_, 10–100 ms), and free water (T_24_, 100–1000 ms) [34]. Each surimi gel sample displayed a similar water distribution profile, with T_23_ as the predominant peak. Nevertheless, some variations were identified among surimi gels with different structures.

**Figure 5:**
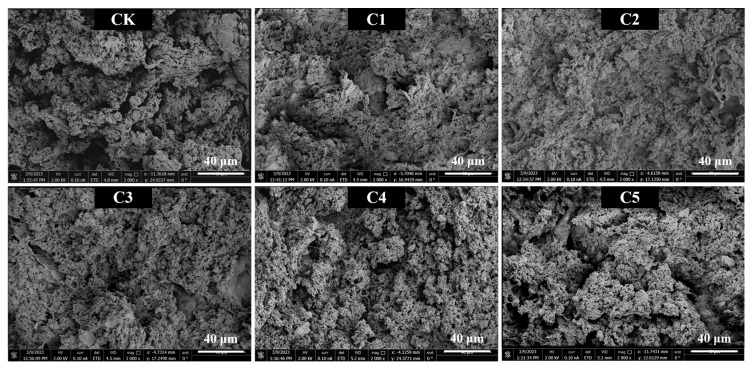
SEM micrographs of novel marine surimi gel samples with different curdlan contents. CK represents the novel marine surimi gel containing egg-white protein; C1, C2, C3, C4, and C5 represent the novel marine surimi gels in which egg-white protein is replaced by 0.2%, 0.4%, 0.6%, 0.8%, and 1.0% curdlan, respectively.

Figure 4b illustrates the proportional distribution of different water peak areas in the novel marine surimi gel samples. The P_23_ and P_24_ percentages in the CK group were 87.86% and 8.59%, respectively. The curdlan addition significantly inhibited the conversion of immobilized water (P_23_) to free water (P_24_) in surimi gels. The P_23_ values in all curdlan-added systems exceeded those observed in the CK group, whereas the P_24_ values were lower. The elevated proportion of immobilized water indicates that water migrates from the exterior to the gel network structure of the protein [35]. As the curdlan concentration increased to 0.4%, P_23_ and P_24_ reached their maximum and minimum values, respectively, indicating that incorporating 0.4% curdlan significantly enhanced the binding strength between water and proteins. This promoted the formation of a denser three-dimensional gel network structure (Figure 5, C2 group) and inhibited the migration of water molecules. Hu et al. [17] observed a similar phenomenon upon incorporating curdlan into whey protein isolate.

The T_2_ relaxation time reflects the degree of freedom and binding force of hydrogen protons. Variations in this parameter can reflect the interaction between a substance and its surrounding chemical environment, with shorter relaxation times corresponding to faster molecular exchange, lower degrees of freedom, and stronger binding interactions [19]. Table 2 shows that, compared to the CK group, the T_21_, T_22_, and *T*_23_ relaxation times in surimi gels were reduced upon the addition of curdlan at concentrations of 0.2%–0.8%. The most pronounced reduction (*p* < 0.05) occurred in the C2 group (0.4% curdlan). This suggests that the mobility of water molecules within the surimi structure decreased, and the interaction between water molecules and surimi polymers was strengthened after curdlan treatment. Zhao, Zhou, Xu, Zeng, and Xing [36] investigated how carrageenan and curdlan influence water distribution in low-salt chicken sauce and found that both additives led to shortened T_21_, T_22_, and T_23_ relaxation times, indicating a reduced water mobility within the system. This corresponds to an improved fracture strength and WHC in heat-treated surimi gels (Figure 1b). However, when the added amount of curdlan exceeded 0.8%, the *T*_21_ relaxation time in surimi gels became significantly longer, while T_24_ decreased, indicating an enhanced water mobility. This could be attributed to excessive absorption and swelling of curdlan, which led to an increased distance between protein molecules, enabling more water molecules to exist in a free state.

### 2.7. Microstructure

To help elucidate changes in the gel structure, the distribution of curdlan within the matrix of the novel surimi gel samples was analyzed using SEM. As shown in Figure 5, the three-dimensional network structure of the surimi gel in the CK group had a rough and loose appearance, with abundant and densely distributed water channels, further confirming the low fracture strength and poor WHC of the CK group (Figure 1b). Upon the incorporation of curdlan, the three-dimensional network structure of the surimi gel matrix transformed into a denser and more compact formation, with fewer water channels and an enhanced internal connectivity observed in the figure. When the added amount of curdlan reached 0.4% (C2 group), the three-dimensional network structure of the surimi gel became the densest, featuring the smallest and most evenly distributed water channels. This enhancement in microstructure may be attributed to the excellent gelling properties of curdlan and its ability to modify the intermolecular interactions among proteins during gelation [17]. Moreover, polysaccharides contribute to moisture stabilization and minimize water channels within the gel network, resulting in a more compact and cohesive structure [37]. However, when the curdlan concentration exceeded 0.4% and increased to 1.0%, the microstructure of the novel surimi gel became progressively irregular, characterized by the formation of larger cluster aggregates (C3–C5 group). This irregularity is primarily attributed to the excess curdlan forming a considerable amount of water gels through hydrogen bonding during the heating process. The formed structures significantly hinder the crosslinking and aggregation of MP via hydrophobic interactions, ultimately resulting in block aggregation and coalescence within the gel network [38].

## 3. Conclusions

Curdlan showed the ability to inhibit lipid oxidation, with 0.4% curdlan reducing the TBARS value of surimi gel to its lowest level. As the added curdlan amount increased, the fracture strength, WHC, hardness, and chewiness of the novel surimi gel were significantly improved; however, negative effects were observed when the added amount exceeded 0.4%. This is because excess curdlan absorbed more water for gelation, which limited the formation of the MP network in the surimi gel and weakened the catalytic effect of TGase. At a 0.4% concentration, curdlan addition resulted in significantly enhanced L* and whiteness values of the surimi gel, promoted the crosslinking within surimi, and increased the resistance to high shear stress. Dynamic temperature sweep analysis revealed that the curdlan-added systems exhibited significantly higher G’ values than the CK group. These values initially increased with the added curdlan concentration, reaching a peak at a 0.4% concentration before gradually decreasing. This finding further supports the idea that curdlan enhanced the molecular interactions within the system. Moreover, curdlan addition reduced the critical temperature at which the G’ value in surimi reached its minimum, suggesting a decrease in the thermal denaturation temperature of myosin light chains. Curdlan also significantly shortened the T_21_, T_22_, and T_23_ relaxation times, reducing the mobility of water molecules in the surimi gel, resulting in a denser, more compact gel matrix with fewer “water channels” and enhanced internal connectivity, especially at 0.4% added concentration. However, excessive curdlan levels (0.6–1.0%) led to a significant decline in the performance of the novel surimi gel, causing an irregular microstructure, the formation of larger cluster-like aggregates, and negative effects on the color. In conclusion, this study introduces a new technique to improve the performance of low-salt surimi gel, promoting the sustainable development of the surimi processing industry.

## 4. Materials and Methods

### 4.1. Materials

The experimental materials included marine surimi (with a lipid content of 6.8%), Curdlan (CG-B type, purity > 99%), and TGase (100 IU/g enzyme activity), both sourced from Jiangsu Yiming Biological Co., Ltd. (Taixing, China). The marine surimi was prepared from mixed marine fish species captured in a single net haul, a typical practice in industrial production. As a result, the species composition and proportions were not fixed. To minimize variability, all surimi used in this study was obtained from the same production batch. To protect the MP during frozen storage, the surimi was pretreated with 4.0% sucrose and 4.0% sorbitol as cryoprotectants, following standard low-temperature preservation protocols.

Lys (with a purity of at least 99%) was purchased from Shanghai Yuanye Biotechnology Co., Ltd. (Shanghai, China). Egg-white protein was provided by Henan Wanbang Chemical Technology Co., Ltd. (Zhengzhou, China). All other reagents, procured from Shanghai Sangon Bio-Technology Co., Ltd. (Shanghai, China), were of analytical grade. The components of the novel marine surimi gel—formulated as a low-salt surimi system incorporating both TGase and Lys—are presented in the table below. CK represents the novel marine surimi gel containing egg-white protein, while C1, C2, C3, C4, and C5 represent the novel marine surimi gels in which egg-white protein is replaced by 0.2%, 0.4%, 0.6%, 0.8%, and 1.0% curdlan, respectively.

### 4.2. Preparation of Mixed Surimi Samples

Initially, frozen marine surimi, stored at −80 °C, was thawed at 4 °C for 8–10 h. Subsequently, a 300 g portion of the surimi sample was transferred to a meat grinder and chopped for 1 min without additives. Then, 0.5% salt was incorporated and mixed for 2 min. The necessary additives (TGase, Lys, egg-white protein, and curdlan) were then added, and the mixture was further blended for 5 min, ensuring that the temperature remained below 10 °C throughout the process. The prepared surimi was transferred into a specific mold (50 mm × 20 mm) for shaping and underwent a two-stage water bath heating process: First, the surimi was heated at 40 °C for 30 min, followed by heating at 90 °C for 20 min. Afterward, it was rapidly cooled in ice water for 1 h and then stored overnight in a refrigerator at 4 °C for further use (Table 3).

### 4.3. Evaluation of Lipid Oxidation

Lipid oxidation in the surimi gel system was assessed using the thiobarbituric acid-reactive substance (TBARS) method, as outlined by Luo et al. [39]. The findings were reported as milligrams of malonaldehyde (MDA) equivalents per kilogram of surimi gel.

### 4.4. Determination of Fracture Strength

The fracture strength of each group of surimi gel samples, shaped as 2 cm × 2 cm × 2 cm cubes, was evaluated at room temperature utilizing a TA-XT Plus texture analyzer from Stable Micro Systems Ltd. (Surrey, UK), equipped with a cylindrical probe (P/0.5) for puncture testing. According to the procedure outlined by Fang et al. [30], the specific test parameters were set as follows: pre-test distance of 30 mm; pre-test, test, and post-test speeds each set to 1 mm/s; compression to 30% of the total height of the surimi gel sample; trigger force of 5 g; data acquisition rate of 400 p/s.

### 4.5. Determination of Water-Holding Capacity (WHC)

The surimi gel sample was formed into a cylindrical shape weighing approximately 2 g, with its mass accurately measured and recorded as *M*_1_. It was then wrapped in a double layer of filter paper, placed inside a 50 mL centrifuge tube, and subjected to centrifugation at 3000× *g* for 10 min. Following centrifugation, the sample was removed, re-weighed, and its new mass was recorded as *M*_2_. The WHC values were determined according to the equation:

WHC (%) = *M*_2_/*M*_1_ × 100, (1)

### 4.6. Texture Profile Analysis (TPA)

Following the method established by Gao et al. [2], TPA was employed to measure the hardness, springiness, cohesiveness, chewiness, and resilience of the marine surimi gels. Recognized for its effectiveness in assessing food texture, TPA was conducted using a texture analyzer (TA-XT Plus, Stable Micro Systems Ltd., Surrey, UK) fitted with a cylindrical P/75 probe (75 mm in diameter). This setup simulated the human oral chewing process to generate texture characteristic values aligned with sensory evaluation. The parameters employed for assessing the physical properties were set as follows: a downforce of 5 g, a compression level of 50%, along with pre-test, test, and post-test speeds of 1.0 mm/s.

### 4.7. Color

The color parameters of marine surimi gels, including a* (redness/greenness), b* (yellowness/blueness), and L* (lightness), were determined using a colorimeter (CM-5, Konica Minolta Sensing, Inc., Tokyo, Japan) [29]. Surimi samples from various treatment groups were sliced into 1 cm-thick pieces. The whiteness of the surimi gels was subsequently determined using the following equation:

(2) $$Whiteness=100-\sqrt{{\left(100-L^{*}\right)}^{2}+a^{*2}+b^{*2}},$$

### 4.8. Rheological Properties

The rheological properties of novel marine surimi sol samples were evaluated through steady shear and temperature sweep tests using a Haake–Mars 60 dynamic rheometer (Thermo Scientific, Karlsruhe, Germany) equipped with a P35/Ti probe. Approximately 2 g of surimi samples, subjected to various treatments, was carefully positioned between two parallel plates with a 1 mm gap and sealed with silicone oil to prevent moisture loss. Steady shear tests were conducted following the procedure described by Ma et al. [40]. Prior to the measurement, the surimi samples were equilibrated at 4 °C for 3 min. Shear stress was recorded at shear rates ranging from 0.1 to 100 s^−1^, with a controlled strain of 2% and a frequency of 100 mHz.

Temperature sweep tests of the novel marine surimi samples were carried out in CD-AutoStrain oscillation mode at a controlled strain of 0.02 and a constant frequency of 0.1 Hz. Additional measurement parameters included a temperature scanning range from 20 to 90 °C and a programmed heating rate of 1 °C/min [40].

### 4.9. Low-Field Nuclear Magnetic Resonance (LF–NMR)

Approximately 2 g of a surimi gel sample was shaped into a rectangular shape and left at room temperature for 30 min. It was then wrapped in cling film and carefully inserted into a cylindrical NMR tube with a 15 mm diameter. The measurements were performed using an NMR analyzer (PQ001-20-025V, Niumag Analytical Instrument Co., Ltd., Suzhou, China) employing the Carr–Purcell–Meiboom–Gill (CPMG) sequence to collect T_2_ signals. The peak ratios of the T_2_ and T_2_ relaxation times were obtained through data inversion. The relevant parameter settings included a sampling frequency of 200 kHz, a 90° pulse width of 7.00 μs, a 180° pulse width of 13.52 μs, an echo time of 0.3 ms, and a number of accumulations set to 8 [34].

### 4.10. Scanning Electron Microscopy (SEM)

Following the procedure outlined by He et al. [41], the novel surimi gel samples were cut into small cubes measuring 4 mm × 4 mm × 4 mm. To fix the sample structure, they were immersed in a 2.5% glutaraldehyde buffer (pH 7.2) for 4 h, then rinsed three times with a 0.1 mol/L phosphate buffer (pH 7.2). After that, the samples were subjected to gradient dehydration using ethanol solutions with increasing concentrations (30%, 50%, 70%, 90%, 95%, 100%), each for 30 min, followed by washing three times with tert-butanol for 30 min each. Freeze-drying was then performed. The prepared surimi gel samples were mounted on stubs, coated with a thin layer of gold via sputtering, and analyzed using a high-resolution field-emission scanning electron microscope (Verios 460, FEI Inc., Hillsboro, OR, USA).

### 4.11. Statistical Analysis

Experimental data were analyzed using one-way analysis of variance (ANOVA) in SPSS 23.0. Post-hoc analysis was carried out using the LSD pairwise multiple comparison method, with *p* < 0.05 indicating significant differences. The data were presented as mean ± standard deviation (SD). All parallel groups were prepared from the same batch of surimi to ensure consistency across treatments. Each analysis was conducted in triplicate (*n* = 3), with three parallel samples measured for each group. Graphs were created using the SigmaPlot 15.0 software.

## Figures and Tables

**Table 1:** Effects of curdlan concentration on textural properties of novel marine surimi gel.

Sample	Hardness (g)	Springiness	Cohesiveness	Chewiness (g)	Resilience
CK	2186.28 ± 166.61 ^e^	0.86 ± 0.01 ^ab^	0.72 ± 0.01 ^a^	1382.40 ± 51.96 ^e^	0.40 ± 0.01 ^ab^
C1	2280.34 ± 97.16 ^d^	0.86 ± 0.02 ^ab^	0.72 ± 0.00 ^a^	1436.89 ± 63.61 ^de^	0.39 ± 0.00 ^bc^
C2	2903.45 ± 91.94 ^a^	0.88 ± 0.01 ^a^	0.72 ± 0.01 ^a^	1781.44 ± 43.94 ^a^	0.41 ± 0.01 ^a^
C3	2672.97 ± 126.53 ^b^	0.87 ± 0.03 ^ab^	0.73 ± 0.01 ^a^	1677.84 ± 70.76 ^b^	0.40 ± 0.01 ^ab^
C4	2681.43 ± 85.23 ^b^	0.86 ± 0.01 ^ab^	0.71 ± 0.03 ^ab^	1568.83 ± 72.02 ^bc^	0.38 ± 0.00 ^cd^
C5	2396.71 ± 73.01 ^c^	0.85 ± 0.04 ^b^	0.69 ± 0.02 ^b^	1510.73 ± 86.14 ^cd^	0.37 ± 0.00 ^d^

CK represents the novel marine surimi gel containing egg-white protein; C1, C2, C3, C4, and C5 represent the novel marine surimi gels in which egg-white protein is replaced by 0.2%, 0.4%, 0.6%, 0.8%, and 1.0% curdlan, respectively. Different letters (a–e) marking the same parameter group indicate significant differences (*p* < 0.05).

**Table 2:** Effects of curdlan concentration on T_2_ relaxation time in novel marine surimi gels.

Sample	T_21_ (ms)	T_22_ (ms)	T_23_ (ms)	T_24_ (ms)
CK	0.425 ± 0.077 ^ab^	1.956 ± 0.335 ^a^	47.686 ± 1.361 ^a^	310.787 ± 7.953 ^c^
C1	0.370 ± 0.106 ^b^	1.825 ± 0.104 ^ab^	44.488 ± 2.183 ^b^	357.079 ± 11.582 ^a^
C2	0.322 ± 0.041 ^b^	1.482 ± 0.144 ^b^	44.488 ± 2.206 ^b^	357.079 ± 9.367 ^a^
C3	0.425 ± 0.113 ^ab^	1.825 ± 0.180 ^ab^	47.686 ± 1.859 ^a^	357.079 ± 8.116 ^a^
C4	0.370 ± 0.083 ^b^	1.589 ± 0.226 ^ab^	44.488 ± 2.667 ^b^	357.079 ± 5.219 ^a^
C5	0.561 ± 0.094 ^a^	1.659 ± 0.083 ^ab^	44.488 ± 1.407 ^b^	333.129 ± 13.823 ^b^

CK represents the novel marine surimi gel containing egg-white protein; C1, C2, C3, C4, and C5 represent the novel marine surimi gels in which egg-white protein is replaced by 0.2%, 0.4%, 0.6%, 0.8%, and 1.0% curdlan, respectively. Different letters (a–c) marking the same parameter group indicate significant differences (*p* < 0.05).

**Table 3:** Marine surimi samples obtained by different treatments.

Sample	Surimi (g)	TGase (w/w, %)	Egg-White Protein (w/w, %)	Curdlan (w/w, %)	Lys (w/w, %)	NaCl (w/w, %)	Cold Water (%)
CK	300	0.4	7.0	–	0.2	0.5	3
C1	300	0.4	0	0.2	0.2	0.5	3
C2	300	0.4	0	0.4	0.2	0.5	3
C3	300	0.4	0	0.6	0.2	0.5	3
C4	300	0.4	0	0.8	0.2	0.5	3
C5	300	0.4	0	1.0	0.2	0.5	3

## Data Availability

The original contributions presented in this study are included in the article. Further inquiries can be directed to the corresponding authors.
